# Engaging with faith communities to tackle ethnic health inequalities in the UK: a scoping review

**DOI:** 10.1136/bmjph-2025-003816

**Published:** 2026-01-27

**Authors:** Datapwa Mujong, Poppy Angelica Spaceman Pierce, Stuart Andrew Green Hofer, Leonora G Weil, Roxanne Crosby-Nwaobi, Jenny Husbands, Richard Antony Powell

**Affiliations:** 1 Primary Care and Public Health, Imperial College London School of Public Health, London, UK; 2 Medical School, University College London, London, UK; 3 UK Health Security Agency, London, UK; 4 Institute of Ophthalmology, University College London Faculty of Brain Sciences, London, UK; 5 Ethnicity and Health Unit, Imperial College London School of Public Health, London, UK

**Keywords:** Public Health Practice, Scoping Review, Social Medicine, Community Health

## Abstract

**Objectives:**

To explore how faith communities have been engaged in the design or delivery of public health interventions addressing ethnic health inequalities (EHIs) in the UK, the outcomes reported, and the barriers and facilitators influencing engagement.

**Design:**

Scoping review.

**Data sources:**

MEDLINE, Embase, PsycINFO, CINAHL, Web of Science, SCOPUS, Cochrane Library and Healthcare Management Information Consortium were systematically searched. Websites of two leading faith-based organisations—FaithAction and Theos Think Tank—were also hand-searched for grey literature.

**Eligibility criteria:**

UK-based empirical studies (2014–2024 inclusive) reporting faith community engagement (CE) in the design or delivery of public health interventions addressing EHIs or their wider social and structural determinants. Non-empirical studies, and studies with no meaningful involvement of faith communities, were excluded.

**Data extraction and synthesis:**

Two reviewers independently screened and extracted data. A descriptive analytical approach was used to chart faith CE approaches, reported outcomes, and barriers and facilitators.

**Results:**

16 studies were included. Faith CE involved collaborations and partnerships, volunteer or peer roles, and places of worship as community hubs. Public health interventions, primarily health education, were typically delivered at a local scale. Health system involvement varied across studies, encompassing roles in funding, design, delivery and research. Reported outcomes included direct benefits from engagement processes and indirect benefits from interventions, predominantly psychosocial rather than behavioural or structural. Common barriers included limited resources, mistrust, cultural misalignment and unequal power dynamics; facilitators included trust, cultural alignment, supportive leadership and clearly defined roles.

**Conclusions:**

Faith communities are vital partners in tackling EHIs; however, they are currently engaged within a limited scope. Strengthening community-led approaches across health system footprints, addressing power dynamics, and evaluating behavioural, structural, and equity-focused outcomes can enhance their impact. The proposed practical actions provide decision-makers guidance to support inclusive, sustainable, and cost-effective public health interventions tackling EHIs.

**WHAT IS ALREADY KNOWN ON THIS TOPIC**

- Faith communities are known actors in the advancement of health equity, but little is known of their engagement in UK public health interventions addressing ethnic health inequalities over the last decade.

**WHAT THIS STUDY ADDS**

- Approaches that are not community-led are most often used in the literature, with public health interventions often limited to health education, and outcomes being predominantly psychosocial.
- Cross-cutting barriers and facilitators relevant for future interventions were identified, including trust, cultural alignment, capability, resources and power dynamics.

**HOW THIS STUDY MIGHT AFFECT RESEARCH, PRACTICE OR POLICY**

- Primary research should explore a wider range of public health interventions and outcomes, diverse approaches to engaging faith communities, and associated barriers and facilitators in-depth.
- Secondary syntheses identifying what works, for whom and why could also guide more targeted and effective practice.
- Multisectoral collaborations are needed to implement the practical actions proposed, addressing barriers and embedding facilitators across local, regional and national health system footprints.

## Introduction

A broad spectrum of ethnic health inequalities (EHIs) persists throughout the life course in the UK,^1^ including infant mortality,^2^ childhood obesity,^3^ adult cardiometabolic disease,^4 5^ vaccine and screening uptake,^5 6^ several mortality outcomes^2 5^ and self-reported health-related quality of life.^5^ While socioeconomic deprivation plays an important role, these disparities are fundamentally shaped by structural racism and discrimination.^1^ Public health systems can inadvertently uphold these power imbalances,^4 7^ producing interventions that are often mistrusted by minoritised ethnic groups and failing to address EHIs meaningfully.

Community engagement (CE) is recommended as an effective strategy to reduce health inequalities as it addresses the structural marginalisation and mistrust underpinning these disparities.^8^,^10^ CE encompasses a continuum of different approaches aimed at maximising the involvement of local communities in shaping health and well-being initiatives.^10^ While international evidence demonstrates the effectiveness of CE in improving health outcomes among minoritised ethnic groups,^11^ limited evidence exists of their impact or barriers and enablers for the UK’s distinct minoritised ethnic groups.^12^

Given that 81% of UK minoritised ethnic groups identify with a religion,^13^ and faith groups were critical in addressing ethnic inequities in COVID-19 vaccine uptake,^14^,^16^ faith CE has gained increasing significance in tackling EHIs.^15 17 18^ This review adopts a definition of faith community as groups of people sharing a common faith or religion.^10^ This encompasses faith leaders, congregations, organisations and networks. It does not refer to assets such as buildings. Faith CE refers to approaches aimed at maximising the involvement of faith communities in public health interventions^10^ and can be classified as collaborations and partnerships, volunteer and peer roles, access points to community resources and strengthening communities.^8^

Faith communities operate within complex systems and hold a wealth of physical, social and cultural assets that have long contributed to health promotion and the mitigation of wider determinants of health.^19^,^21^ However, engagement has not been without past challenges, and tensions have surfaced when public health approaches and faith-based agendas diverged or were misaligned.^19 22 23^ The COVID-19 pandemic highlighted existing health inequalities among ethnic and religious minorities but also advanced the faith-health agenda, exemplified by initiatives such as the WHO Faith Network and other effective approaches in the UK to reducing health disparities.^15^ In this way, the pandemic acted as a catalyst for collaborations between public health bodies and faith communities, enabling the support of accessible care pathways, alignment of religious practice with public health efforts and sharing of trusted culturally relevant health information,^14^,^1624 25^ some of which have continued beyond that crisis.^15 17^

Recognising the benefits of engaging faith communities to support good health, particularly in minoritised ethnic groups,^1519^,^21 25^ and its increasing prominence in UK policy focus,^15 18 26^ there is a pressing need for a robust evidence base^27^ to inform how faith CE can be effectively supported, integrated and sustained to reduce EHIs. The most recent and comprehensive UK-focused literature review, published by FaithAction in 2014,^20^ identified only three studies evaluating public health interventions involving faith communities and minoritised ethnic groups. The review found initiatives targeting smoking cessation, diabetes awareness and general health promotion, primarily delivered through faith leaders or faith settings. While the review highlighted the potential of diverse engagement models, it found that evaluations were largely descriptive, methodologically weak and offered limited insight into implementation.^20^

This review explores the UK literature since the FaithAction review in 2014,^20^ capturing faith CE since its recent policy prominence. It specifically seeks to explore in further detail the range of faith CE approaches in UK-based public health interventions to address EHIs, what outcomes have been reported, and what factors have influenced faith CE, as these have yet to be comprehensively synthesised to our knowledge. Some inferences can be drawn from a scoping review on obesity prevention undertaken in UK Islamic settings^28^; however, its scope is limited. A 2016 scoping review of all types of CE in the UK,^29^ and an associated 2015 review of their barriers and facilitators,^30^ further revealed a dearth of literature on faith groups and did not distinguish findings relevant to faith communities or minoritised ethnic groups.

A scoping review methodology is appropriate for this review given the heterogeneity of faith CE approaches, the range of barriers and facilitators, and the likelihood of methodological inconsistency across the literature. The potential outcomes also need to be mapped to assess if types of outcomes decision-makers need—including impact on equity or structural outcomes—are studied. Scoping reviews are designed to map the extent, nature and characteristics of available evidence and identify conceptual and empirical gaps.^31^ This scoping review provides an overview that informs policy and future research.

### Objectives

This review explores how faith communities have engaged (ie, approaches) in the design or delivery of public health interventions addressing EHIs in the UK, the types of health or social outcomes that have been reported from these interventions, and the barriers and facilitators to engaging faith communities.

## Methods

This review follows the updated methodological guidance from the Joanna Briggs Institute (JBI) on data extraction, analysis and presentation of results.^32^ Two reviewers (DM and PASP) conducted all stages of this scoping review, which is reported following the PRISMA-ScR guidelines for systematic reporting.^33^

### Eligibility criteria

Records were screened based on predefined eligibility criteria detailed in online supplemental appendix 1. Only empirical studies, including qualitative and quantitative ones, that engaged any faith community in the design or delivery of public health interventions were included. Non-empirical studies, purely descriptive studies and abstracts without full texts were excluded. EHIs were defined as systematic, avoidable and unfair differences in health or healthcare between ethnic groups.^2^ Eligible outcomes included physical or mental health, behavioural indicators, uptake of preventive services (eg, screening, vaccination) and relevant social or structural proxies, such as discrimination, trust in healthcare or social support. Outcomes could be for indirect beneficiaries, defined as those minoritised ethnic groups receiving the public health intervention or for direct beneficiaries, defined as those faith community actors involved in the CE approaches.

We also included studies exploring experiences, including barriers and facilitators, of engaging faith communities in designing or delivering public health interventions. Only studies in English and studies based in the UK (England/Wales/Scotland/Northern Ireland; including devolved administrations and local authority programmes) were included.

### Search strategy

The authors searched relevant peer-reviewed medical databases (ie, MEDLINE, Embase, PsycINFO through OVID, CINAHL through EbscoHost and Web of Science), a social sciences database (SCOPUS-all), and the Cochrane Library database. References of included studies and systematic reviews were searched to identify missed studies. A grey literature search included the Healthcare Management Information Consortium (HMIC) and a hand search of other grey literature from leading faith-based organisations (ie, FaithAction and Theos Think Tank).

Online supplemental appendix 2 shows the search strategy for bibliographic databases. It was developed and tailored to the research aim, including terms related to faith, ethnicity, public health and the UK. The search dates were limited to from June 2014, the search date of the last relevant comprehensive review,^20^ to August 2024 inclusive.

### Screening process

All search results were uploaded to EndNote to remove duplicates and manage references. Records were then exported to Covidence, where their titles and abstracts were screened against the eligibility criteria, followed by full-text screening. Screening was carried out independently by two reviewers (DM and PASP). Conflicts were reviewed in team meetings and studies were only included if there was full agreement among the authors.

### Data charting

A structured data extraction template (online supplemental appendix 3) was developed and piloted based on the JBI guidance. Data were extracted deductively on study characteristics, public health topic, details of faith CE, target population, public health intervention details and outcomes reported. Open coding of extracted data on factors affecting implementation was completed on Microsoft Excel independently (by DM and PASP) to avoid missing new concepts unique to this field. These were discussed in iterative meetings, and for a small number of codes where conflict arose, consensus discussions took place (SAGH) and codes were revised. A framework of categories was developed for barriers and facilitators, and when agreed (DM, PASP and SAGH), DM and PASP organised relevant information within the framework. This was reviewed by the team in further discussions until categorisations were deemed appropriate. In multipaper interventions, we only reported findings once if they were repeated.

### Data synthesis

Data were synthesised using a descriptive analytical approach in line with the JBI scoping review methodology. Extracted data were collated and organised to identify patterns across studies. Faith CE approaches were classified according to a public health ‘community-centred approaches’ framework as: collaborations and partnerships, volunteer and peer roles, access points to community resources and strengthening communities.^8^ Data on implementation were inductively coded and summarised into key barriers and facilitators. These were subsequently compared against existing literature to help contextualise the findings and highlight policy-relevant factors. Outcomes were grouped according to their nature (eg, behavioural, psychosocial, structural determinants of health), with attention to both direct and indirect beneficiaries.

### Critical appraisal

Critical appraisal was not completed in this study, in alignment with the JBI guidelines.

### Patient and public involvement

As this work was completed as part of an academic award, there were no opportunities to involve patients or members of the public in the review. However, the research team recognises the sensitive nature of this work and includes individuals drawn from various minoritised ethnic groups.

## Results

### Selection of included studies

**Figure 1:**
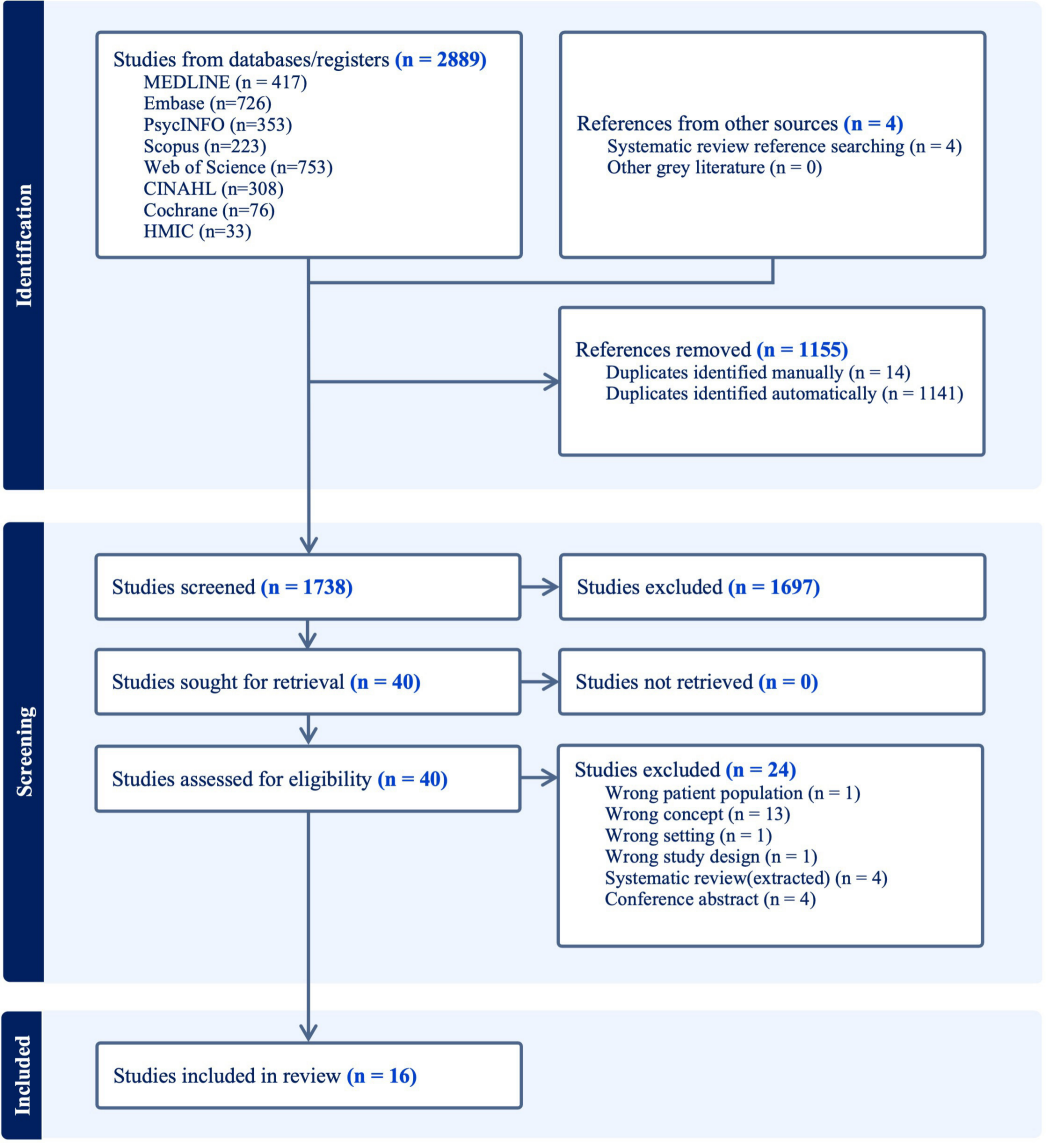
PRISMA flow diagram. HMIC, Healthcare Management Information Consortium; PRISMA, Preferred Reporting Items for Systematic Reviews and Meta-Analyses.

After screening 1738 titles and abstracts, 40 full-text reports were retrieved. Of these, 16 studies met the eligibility criteria for inclusion.^1634^,^48^ A PRISMA flow diagram presents this process in figure 1.

### Characteristics of included studies

As displayed in table 1, this review included 16 studies^1634^,^48^ published between 2015 and 2024, all conducted in the UK, with most based in England and three in Scotland.^38 44 47^ Study designs were diverse: eight used qualitative methods,^3536 38^,^40 43 44 47^ three employed before-and-after designs,^34 45 48^ three were mixed-methods studies^37 41 46^ and one used a cross-sectional survey design.^16^ Only one study was a randomised controlled trial.^42^ There were 6 studies published pre-2020^40^,^4346 48^ and 10 published post-2020.^1634^,^39 44 45 47^

**Table 1:** Core characteristics of studies included in the scoping review

Study (author, year)	Study design	Faith community	Minoritised ethnic groups (demographics)	Ethnic health inequality	Community engagement approach	Public health intervention (geography)	Health service organisations involved	Health service roles
Waqar *et al*, 2024^34^	Before-and-after	Muslim	Asian (adults, mixed sex)	Cancer Screening	Collaboration and partnership; Volunteer and peer roles; place of worship	Health education: Bowel cancer screening (Local- East London)	North East London Cancer Alliance (NHS)	Funding, design, research
Kasstan *et al*, 2023^36^	Qualitative	Jewish	Jewish (children and adults)	Infectious disease	Collaboration and partnership.	Health education: Polio vaccine (Regional–London)	UK Health Security Agency, NHS, Local public health teams	Design, delivery
Codjoe *et al*, 2023^37^	Mixed methods	Christian	Black (adults, mixed sex)	Other mental health	Collaboration and partnership; volunteer and peer roles.	Health education: mental health awareness (Local–South London)	NHS Foundation Trust	Design, delivery, research
Christie-de Jong *et al*, 2022^38^	Qualitative	Muslim	Asian; other ethnic group: Arab (adult women only)	Cancer screening	Collaboration and partnership; volunteer and peer roles;	Health education: Breast, colorectal and cervical cancer screening (Local–Glasgow)	Not specified	Not specified
Kasstan *et al*, 2022^35^	Qualitative	Jewish	Jewish (mixed sex)	Infectious disease	Volunteer and peer roles;	Vaccine delivery; Health education: COVID-19 vaccine (Local–North London)	Public Health England, NHS facilities	Funding, design, delivery, research
Darko *et al*, 2020^39^	Qualitative	Muslim	Asian (mixed sex)	Diabetes	Collaboration and partnership; volunteer and peer roles;	Health education: Diabetes in Ramadan; primary care delivery. (Regional–East Midlands)	NHS general practices, clinical commissioning group	Delivery
King *et al*, 2017^40^	Qualitative	Muslim	Asian (children and adults)	Smoking	Collaboration and partnership; volunteer and peer roles; place of worship	Health education: Smoke Free programme (Regional–Birmingham, Bradford, Leeds)	Not specified	Not specified
Maynard *et al*, 2017^41^	Mixed methods	Christian, Muslim, Hindu	Asian; Black (children)	Obesity; diet; physical activity	Collaboration and partnership; volunteer and peer roles; place of worship	Health education: obesity, diet, physical activity (Local–inner city London)	Not specified	Not specified
Shah *et al*, 2015^42^	Randomised Controlled Trials	Muslim	Asian (children and adults)	Smoking	Collaboration and partnership; Volunteer and peer roles; place of worship	Health education: Smoke Free programme (Regional–Birmingham, Bradford, Leeds)	Not specified	Not specified
Mantovani *et al*, 2017^43^	Qualitative	Christian	Black (adults)	Other mental health	Collaboration and partnership; volunteer and peer roles; strengthening communities	Health education: Mental health (Local–South London)	Local public health team, NHS Foundation Trust	Commissioning, funding, design.
Ali *et al*, 2022^45^	Before-and-after	Muslim	Asian; Other ethnic group: Arab (mixed gender)	Other: organ donation	Collaboration and partnership; volunteer and peer roles; Place of Worship	Health education: organ donation (National–Glasgow, Leeds, London, Manchester, Newcastle, Nottingham and Bradford)	NHS Blood and Transplant	Funding
Hall *et al*, 2024^44^	Qualitative	Muslim	Asian (adults)	Obesity	Collaboration and partnership; volunteer and peer roles;	Health education: Toolkit for obesity prevention (Local–Bradford)	Not specified	Not specified
Hankir *et al*, 2017^48^	Before-and-after	Muslim	Asian (adults)	Other mental health	Collaboration and partnership; volunteer and peer roles;	Health education: mental health (Local–Birmingham)	Not specified	Not specified
Kotzur, 2023^47^	Qualitative (Participatory)	Muslim	Asian (adult women only)	Cancer	Collaboration and partnership; volunteer and peer roles;	Health education: Breast, colorectal and cervical cancer screening (Local–Glasgow)	Not specified	Not specified
Perry *et al*, 2018^46^	Mixed methods	Jewish	Jewish (adults)	Other mental health	Collaboration and partnership; volunteer and peer roles;	Health education: mental health (Local–Hackney, London)	NHS Trust, Clinical Commissioning Group	Funding, design, research
Wehling *et al*, 2024^16^	Cross-sectional	Not specified (Mosque-based)	Asian; black; mixed ethnic group; other White; other ethnic group (adults)	Infectious disease	Collaboration and partnership; volunteer and peer roles; place of worship	Vaccine delivery: COVID-19 vaccine (Local–Woking, Surrey)	Public Health England, NHS Trust, Integrated Care System, Health and Care Partnership	Funding, design, delivery

NHS, National Health Service.

Faith communities engaged included Muslim,^1634 37^,^42 44 45 47 48^ Christian^37 41 43^ and Jewish groups.^35 36 46^ Public health interventions were mostly around health education on mental health,^37 43 46 48^ cancer screening,^34 38 47^ vaccines^35 36^ and lifestyle-related conditions, such as smoking^40 42^ and obesity.^41 44^ Two studies had vaccine delivery as their main intervention.^16 35^ The majority of interventions were designed or delivered at a local footprint,^3435 37^,^39 41 43^ with four studies^36 39 40 42^ involving regional footprints and one^45^ nationally.

Targeted minoritised ethnic groups included Asian,^1634 37^,^42 44 45 47 48^ Black,^16 37 41 43^ Jewish,^35 36 46^ Other ethnic group: Arab,^38 45^ Mixed ethnic group,^16^ Other White^16^ and Other ethnic group,^16^ with some studies having multiple groups. Sample sizes ranged from fewer than 10 participants^47^ to over 500.^45^ Most studies involved adult participants aged 18 and over, though some included children or mixed-age groups. Two studies focused exclusively on women.^38 47^

### Synthesis of results

The following results are grouped under (1) Faith CE approaches used in public health interventions; (2) Reported outcomes of faith CE in public health intervention and (3) Barriers and facilitators of faith CE in the design and delivery of public health interventions.

#### Faith CE approaches used in public health interventions

Collaborations and partnerships were described in 15/16 studies^1634 36^,^48^ as a form of engagement and varied in structure and scope. Often as a formalised steering group,^37^ the collaborations were between researchers and faith leaders who contributed to decision-making for interventions. Strategic partnerships also emerged between public health actors and faith partners in coalitions like the London Jewish Health Partnership, a multiagency initiative aiming to reduce health inequalities, for example, through the coproduction of culturally sensitive COVID-19 vaccine messaging.^36^ Where reported, the health system actors collaborating in intervention design included a variety of local and national public health bodies, a local NHS foundation trust, integrated care bodies and specialist NHS bodies.^1634^,^37 43 46^ Studies often had a variety of health system actors, and they played other roles, including commissioning, funding and supporting intervention delivery and research outside of collaborations or partnerships.^1634^,^37 39 43 45 46^

Collaborations also occurred directly with faith community members through participatory codesign processes.^38 44 47^ However, the extent of participatory decision-making was rarely specified, and power dynamics were often under-reported. Only one study^43^ clearly aligned with a ‘strengthening communities’ model, where African and Caribbean faith communities identified their own mental health priorities, co-led intervention development and trained community champions to deliver support. These champions embedded delivery within their own faith settings, and a local research assistant contributed to data collection, highlighting strong community ownership and capacity-building.

Volunteer and peer roles were also prominent in 15/16 studies^1634 35 37^,^48^ and typically involved non-professional, community-aligned individuals delivering or promoting interventions. Faith leaders, such as imams^39 40 42^ and pastors,^37^ were frequently positioned as lay health promoters. Other studies recruited community members as facilitators or champions; for example, the Muslim women leading an online codesign session,^47^ or the community champions supporting mental health advocacy.^43^ Muslim health professionals also volunteered to deliver presentations, blending clinical expertise with cultural competence.^34 48^ They ranged from one-off volunteering^16^ to embedded roles developed over time.^43^

Access to community resources was highlighted in 6/16 studies^1634 40^,^42 45^ and was facilitated through places of worship as trusted, accessible hubs for public health delivery. Mosques were often central intervention sites,^3440^,^42 45^ hosting educational sessions and distributing culturally adapted materials on topics such as childhood obesity and secondhand smoke. Similarly, a mosque-based COVID-19 vaccination centre supported by local volunteers was described.^16^ While these settings provided a vital infrastructure for engagement, they were often used alongside other strategies, such as peer facilitation and collaborative design, reflecting the layered nature of faith CE.

#### Reported outcomes of faith CE in public health interventions

Across the studies, a range of psychosocial, behavioural and social outcomes were reported. These were primarily identified among indirect beneficiaries in 9/16 studies,^34^,^3638 42 43 45 46 48^ with five studies reporting quantitatively.^34 42 45 46 48^ In 3/16 studies,^37 43 44^ outcomes were reported among direct beneficiaries, and one study reported quantitatively.^37^

For indirect beneficiaries, psychosocial outcomes were reported in six studies.^34 38 42 45 46 48^ These included increased knowledge, shifts in attitudes and intentions, and greater motivation or confidence. These outcomes were noted in interventions targeting cancer screening,^34 38^ organ donation^45^ and mental health.^46 48^ Statistically significant improvements in knowledge and behavioural intention were also observed in two studies,^34 45^ alongside reductions in stigma in mental health contexts in one study.^48^

Behavioural and social outcomes among indirect beneficiaries were less consistently reported in 5/16 studies^35 36 42 43 48^ and 4/16 studies,^35 37 43 44^ respectively. There was little to no change in smoking-related behaviours in a smoke-free homes trial,^42^ and another study reported lifestyle changes informed by knowledge gained by volunteers during the intervention.^43^ One study highlighted that vaccine uptake remained low despite localised efforts.^36^ Social outcomes included enhanced trust, new social networks and community cohesion, especially where faith communities supported delivery.^35 37 43 44^

Direct beneficiaries, such as faith community members or leaders in volunteer or peer roles, described improvements in their mental health literacy, communication skills, self-confidence and community leadership capacity.^37 43 44^ Those involved in collaborations to codesign reported greater ownership of the intervention and empowerment.^38 44^

Interventions that were culturally and religiously tailored were found to be acceptable and feasible in three studies.^38 45 47^ However, health promotion activities were deemed inappropriate for places of worship in two studies^16 40^ because of misalignment with the sanctity of such settings and prevention of other faith activities occurring concurrently. Likewise, the health education content was also deemed too complex when tailored in one study exploring relationships between spirituality and mental health.^37^

#### Barriers and facilitators of faith CE in design and delivery of public health interventions

As displayed in table 2, several cross-cutting factors recurred across the literature as barriers and facilitators influencing the implementation of faith CE. These include Trust and relationships; Cultural alignment; Capability and motivation; Opportunity and resources and Leadership and power dynamics.

**Table 2:** Barriers and facilitators of faith CE in the design or delivery of public health interventions

Domain	Barriers	Facilitators
Trust and relationships	Trust between mosque committees, faith leaders and researchers took significant time to build.^40 42^ Communication challenges between stakeholders during codesign processes led to tensions.^44^ Volunteers delivering interventions which participants mistrusted.^35^ The COVID-19 pandemic made reaching some faith community gatekeepers challenging.^47^ Irregular attendance and low community participation.^41^	Multichannel recruitment strategies, including phone and mail.^41^ Meeting people ‘where they are’ helped engagement.^16 42 44^ Trusted champions and religious leaders enhanced recruitment.^38^,^40^ Icebreakers and small-group discussions to build rapport.^47^ Previous collaborations helped leverage trust.^35 43^ Faith leaders perceived as authoritative and respected.^40^ Increased public trust in the intervention due to faith group involvement.^35^ Endorsement by public health increased credibility of faith community delivering the programme.^35^ Peer educators and community facilitators enhanced participation.^37 38^
Cultural alignment	Faith community concerns on using faith setting deemed as sacred for health promotion.^16 40^ Language barriers between organisers, attendees and leaders.^39 41^ Lack of interpreters.^40^ Stigma or misconceptions about health topics such as mental health or smoking.^40 43^	Gender-sensitive approaches such as women-only sessions.^38 42 47^ Building cultural competence through understanding the values of communities.^44^ Tailoring content to religious norms and values.^45^ Translated resources and digital tools supported engagement.^47^ Involving community-based researchers enhanced cultural relevance.^44^
Capability and motivation	Volunteers sometimes did not have adequate training and clarity on some parts of their roles.^43 44^ Communities did not understand the research process.^41^ Some places of worship had long travel distances and waiting times for participants in health promotion activities.^16^ Some places of worship had limited space for health promotion activities.^40^ Lack of compensation for efforts of faith leaders.^40^ Lack of motivation to participate as a comparator group.^34^	Training to develop skills including for community champions, delivering vaccines, facilitating groups and delivering health education.^35 40 42 47^ Motivation of volunteers driven by values, lived experience and community development goals.^39 43^ Online or short sessions improved flexibility.^47^ Accessible locations such as local mosques.^16 42^ Use of multifunctional venues with wider community roles.^42^
Opportunity and resources	Some faith settings had pre-existing commitments limiting flexibility.^40^ Lack of staff or researchers from within faith communities.^41^ Volunteers had difficulty affording transport.^39^ Funding constraints and sustainability concerns.^35 39^ Time to recruit and build rapport and recruit between mosque committees.^40 41^	
Leadership and power dynamics	Mosque committees overruled plans despite faith leader agreement.^40^ Power imbalances hindered equal participation of faith leaders.^44 47^ Mosque committee rules constrained women’s involvement.^40^ Resistance from senior faith leaders to faith leaders acting as health promoters.^37^	Supportive and enthusiastic religious leaders enabled engagement.^40 41^ Autonomy for faith leaders to deliver interventions in their own way.^40 42^

CE, community engagement.

#### Trust and relationships

In 6/16 studies,^3540^,^42 44 47^ when trust and good quality relationships between stakeholders were a challenge, they acted as barriers to faith CE. A lack of trust between researchers and religious institutions complicated collaborations,^40 42^ while mistrust of public health interventions, particularly vaccines, undermined volunteer-led delivery.^35^ In coproduction, miscommunication among stakeholders, where power dynamics were not clear, strained partnerships.^44^ Additionally, the COVID-19 pandemic-related disruptions^47^ and irregular community attendance^41^ created further challenges to building sustainable relationships with places of worship.

Building trust and good relationships acted as faith CE facilitators in 11/16 studies.^1635 37^,^44 47^ Multichannel recruitment strategies^41^ and outreach directly to communities^16 44^ helped build relationships, and building on prior collaborations often renewed trust.^35 43^ The use of trusted volunteers and peers^37^,^40^ and interpersonal strategies, such as small-group formats and icebreaker activities, promoted rapport and trust during sessions.^47^ Public health endorsements added legitimacy to volunteers delivering interventions.^35^

#### Cultural alignment

Misalignment between the culture of some faith communities and either CE approaches or interventions posed significant barriers to CE in 5/16 studies.^1639^,^43^ Participants, and some faith leaders, raised concerns about the perceived sanctity of places of worship being compromised by engaging in health promotion activities.^16 40^ Volunteers and collaborations were also hindered by stigma surrounding certain topics like mental health and smoking,^40 43^ and by language barriers among organisers, facilitators and congregants.^39^,^41^

Conversely, cultural sensitivity and tailoring were essential enablers of engagement in 5/16 studies.^38 42 44 45 47^ Gender-sensitive approaches for Muslim women,^38 42 47^ content grounded in religious values^45^ and the provision of translated materials^47^, helped enable collaborations and participation by places of worship. Collaborations were also supported when researchers with shared religious or ethnic backgrounds of participants were engaged.^44^

#### Capability and motivation

In 6/16 studies,^16 34 40 41 43 44^ capabilities and motivation in faith communities acted as a barrier to faith CE. Psychological capability was a challenge when roles were not clear,^43 44^ and some community champions felt they had not been adequately trained to manage some challenges in mental health promotion.^43^ Some faith communities also found participation challenging as they did not understand the research process.^41^ This was complicated by limited compensation for faith communities,^40^ including allocation to comparator arms without receiving any intervention.^34^ The physical capability of places of worship further influenced their engagement. Some lacked adequate space, staff or interpretation services,^40 41^ while logistical barriers, such as long travel distances and waiting times for attendees, created further challenges.^16^

In 7/16 studies,^16 35 39 40 42 43 47^ where capabilities and motivation were addressed, it acted as a facilitator to faith CE. Formal training mitigated some of these gaps and supported implementation,^35 40 42 47^ including training peer educators with group facilitator skills,^47^ religious leaders to deliver health education,^40 42^ faith community members as community champions,^43^ and faith volunteer emergency service as vaccinators.^35^ Volunteers were also more likely to remain engaged when their work was tied to lived experience, intrinsic motivation or community development goals.^39 43^ Physical capability was tackled by using places of worship valued as accessible and multifunctional venues well-suited to community outreach,^16 40^ particularly when paired with flexible formats like shorter sessions or online engagement.^47^

#### Opportunity and resources

Opportunity and resource investment for CE presented consistent challenges across stakeholder groups in 4/16 studies.^3539^,^41^ Health practitioners reported that recruiting faith groups and establishing partnerships required more time than anticipated,^40 41^ while faith communities cited limited availability due to competing obligations or inadequate staffing.^39 40^ Financial constraints, such as lack of travel reimbursement or sustained funding, also further limited volunteer participation.^35 39^

#### Leadership and power dynamics

Power asymmetries, both within faith institutions and between researchers and communities, were a barrier to CE implementation in 4/16 studies.^37 40 44 47^ In some cases, faith committees overruled volunteer plans or imposed constraints on women’s participation.^40^ Faith leaders, although involved in partnerships, sometimes resisted assuming health roles.^37^ Within coproduction efforts, unequal influence or unclear power-sharing arrangements disrupted collaboration.^44 47^ However, in 3/16 studies^40^,^42^ where religious leaders were actively engaged, supportive and given autonomy in delivery, faith CE implementation was more robust.

## Discussion

This scoping review synthesised evidence from 16 UK-based peer-reviewed studies examining faith CE in the design or delivery of public health interventions to tackle EHIs, including their health and social outcomes, and barriers and facilitators. The interventions occurred largely on local footprints, with a variety of faith communities and health system actors involved. Faith CE approaches were categorised under four domains: collaborations and partnerships, volunteer and peer roles, access points to community resources and strengthening communities. These domains were non-mutually exclusive, and a variety of approaches were used concurrently in studies.

Collaborations and partnerships were a common form of engagement for decision-making in 15/16 studies,^1634 36^,^48^ ranging from informal cooperation with local leaders to formal steering groups and faith partnerships. However, the degree of power-sharing in these collaborations was often not detailed. When explored, power struggles were usually described, or leadership appeared to be outside of faith communities.^34 47^ Only one study^43^ exemplified a ‘strengthening communities’ approach in which faith communities led the identification of local mental health needs, employed and trained community champions, and shaped both design and implementation. This finding reinforces concerns raised in public health literature about hierarchical power relationships in faith and health partnerships, with public health and local authorities having control of knowledge and funding.^18 22 49^

Volunteer and peer roles also featured prominently in 15/16 studies,^1634 35 37^,^48^ particularly in interventions leveraging the trust and respect of imams, pastors and lay leaders. These roles were often framed as culturally resonant in health promotion and were instrumental in gaining trust or legitimising interventions.^35 38^ However, the review also highlighted concerns around limited training for community champions in managing complex situations, and unclear role definitions in codesigning and logistical burdens for faith communities, such as funding and resourcing.^39 44^ The wider literature similarly highlights that, while faith leaders can be highly effective health messengers, their involvement requires careful support to avoid tokenism.^17 23^

Six studies^1634 40^,^42 45^ involved the use of places of worship as sites for intervention delivery, reflecting the domain of ‘access to community resources’. This was often part of broader collaborations or support by volunteers from within the faith community.^34 40 42^ Their symbolic and social significance contributed to accessibility, legitimacy and engagement.^16 45^ This supports the existing literature, which challenges the notion of faith settings merely as physical hubs, but as trusted, moral and relational spaces in which health and well-being is coproduced.^50^

Across the included studies, outcomes predominantly focused on psychosocial change among indirect beneficiaries of the public health interventions, including improved knowledge, confidence and health intentions among minoritised ethnic groups.^34 45 48^ Fewer studies examined the direct impacts of engagement on those involved in the CE approaches and reported enhanced agency or leadership confidence.^37 43^ Impact on health behaviours was reported in five studies^35 36 42 43 48^; however, no studies evaluated impacts on wider determinants of health, such as racism or deprivation. This may reflect the nature of most public health interventions, which focused on health education. It, however, also aligns with literature highlighting that evaluations of initiatives involving faith communities are often short-term or focused on surface-level behaviour change.^20^ Additionally, our review did not find studies exploring potential negative impacts or unintended consequences, which has been a concern raised in the literature.^27 29^

The barriers and facilitators to faith CE aligned with factors identified in the broader CE literature. Several mirrored known contextual and infrastructural issues around CE,^30^ including concerns around trust and quality relationships^40 42^ and resistance to power-sharing.^44^ Limited investment in training, resources and inclusive practices, particularly language and cultural tailoring, was also evident,^39 44^ echoing researched themes around capacity and access.^30^ Facilitators often stemmed from building trusting relationships, supportive leaders and coproduction with respected community members.^38 43^ These findings parallel observations that logistical and relational challenges limit engagement efficacy unless met with long-term, trust-based collaboration.^23^ However, in contrast to concerns about bureaucratic inefficiencies,^23^ studies in this review did not describe policy-level misalignments, likely reflecting their limited scope on implementation factors.

### Policy implications

Practical actions for health systems to support faith health collaboration are listed in box 1.

Box 1 **Practical actions to strengthen faith community engagement**

1. Establish equitable, long-term partnerships

Develop formal networks or steering groups connecting faith leaders, commissioners, researchers and public health teams. Define roles and responsibilities early to mitigate power imbalances and ensure co-ownership of decisions.

2. Embed faith engagement within system architecture

Integrate faith community engagement into strategic planning, commissioning and governance structures.

Include clear accountability mechanisms and culturally safe partnership agreements in policy and contracting frameworks.

3. Resource and sustain collaboration

Provide multiyear funding that covers programme delivery, training, infrastructure and relationship-building. Compensate faith partners fairly and support accredited training pathways for volunteers and leaders.

4. Strengthen cultural alignment and codesign

Involve faith communities from inception to evaluation. Coproduce interventions and resources that align with faith values and practices, delivered by culturally and linguistically matched facilitators.

5. Build system capability and readiness

Conduct organisational readiness and cultural safety assessments to ensure health system preparedness to engage equitably with faith groups. Train staff in faith literacy and community partnership working.

6. Coordinate across footprints for equity of reach

Link local efforts to regional and national structures to share learning, avoid duplication and enhance strategic reach—particularly for smaller or dispersed faith groups.

7. Evaluate for impact and equity

Adopt equity-sensitive and structural metrics within evaluation frameworks. Use participatory evaluation to understand how barriers and facilitators shape outcomes and sustainability.

Further research would be needed to understand the relative impact of each of these different actions and the impact on the structural determinants of health.

This review offers valuable insights regarding the geographical footprint of faith-health initiatives. While most studies operated at a local level, four had a regional scope and one functioned nationally. Moreover, local engagement remains crucial for building trust and understanding faith community needs, and most related studies emphasise the importance of the tailored local context.^6^ However, regional and national approaches can add strategic value by enabling the escalation of key issues, sharing of good practice, facilitation of policy change where appropriate, enhancing reach and efficiency, particularly for smaller faith groups or communities that cluster across different regions or across administrative boundaries.^7^

### Limitations

This review has several limitations to be considered in its interpretation. First, we limited the review time frame from 2014 to 2024. The FaithAction review published in 2014 may have missed prior studies as they searched a limited range of sources, and their eligibility criteria were unclear. Second, peer-reviewed, published studies were primarily included despite some literature about CE often appearing in grey literature.^29^ To reduce publication bias, we searched grey literature from the HMIC, FaithAction and Theos Think Tank ; however, and importantly, there was minimal yield.

Third, the studies provided limited detail about the nature of the engagement approaches; therefore, categorising into CE approaches and understanding power dynamics was challenging. Likewise, studies often provided limited insights into outcomes or barriers and facilitators of CE, because their main aim was often assessing for acceptability and feasibility of public health interventions. Fourth, as a scoping review, our analysis focused on highlighting patterns and gaps in the literature and does not offer a comprehensive synthesis of effectiveness or factors influencing implementation. Finally, a quality appraisal of all the studies was not undertaken and so findings must be interpreted accordingly.

## Conclusions

Faith communities continue to play an important, but underleveraged role in addressing EHIs in the UK. Their involvement over the last decade has often been at a local footprint, in health education or service delivery initiatives, through partnerships and collaborations, volunteer and peer roles, and places of worship. However their potential to lead initiatives, transform the structural roots of EHIs such as racism, and reduce EHIs remains underexplored. An evaluative co-ordinated multisectoral approach is therefore needed, with faith community assets being strategically integrated into the health system across local, regional and national footprints to tackle EHIs.

Gaps that should be addressed in faith community engagement, include understanding power dynamics in engagement and community-led approaches from diverse faith groups. A wider range of public health interventions also require evaluation, extending beyond health education to include building healthy public policy, creating supportive environments and reorienting health services. Robust, equity-sensitive evaluations should capture behavioural, social, structural and equity-relevant impacts, going beyond psychosocial measures. Future research could also further build on this review by exploring what aspect of faith CE and different public health interventions work, for which minoritised ethnic groups and subpopulations, and why.

## Supplementary material

10.1136/bmjph-2025-003816 online supplemental appendix 1

10.1136/bmjph-2025-003816 online supplemental appendix 2

10.1136/bmjph-2025-003816 online supplemental appendix 3

## Data Availability

Data are available on reasonable request.

## References

[1] Race Equality Foundation Racism is the root cause of ethnic inequalities in health. https://raceequalityfoundation.org.uk/wp-content/uploads/2023/02/CC167_REF_Briefing_Paper_vs4.pdf.

[2] Esan O, Adjei NK, Saberian S (2023). Mapping Existing Policy Interventions to Tackle Ethnic Health Inequalities in Maternal and Neonatal Health in England: A Systematic Scoping Review with Stakeholder Engagement.

[3] Public Health England Differences in child obesity by ethnic group.

[4] Robertson R, Williams E, Buck D (2021). Ethnic Health Inequalities and the NHS: Driving Progress in a Changing System.

[5] Raghib A, Avirup C, Nita F (2021). Ethnic Disparities in the Major Causes of Mortality and Their Risk Factors – a Rapid Review.

[6] UK Health Security Agency (2025). Health inequalities in health protection report 2025.

[7] Selvarajah S, Corona Maioli S, Deivanayagam TA (2022). Racism, xenophobia, and discrimination: mapping pathways to health outcomes. The Lancet.

[8] Public Health England (2015). A Guide to Community-Centred Approaches for Health and Wellbeing.

[9] World Health Organization (2020). Community engagement: a health promotion guide for universal health coverage in the hands of the people. https://www.who.int/publications/i/item/9789240010529.

[10] National Institute for Health and Care Excellence (2025). Community engagement: improving health and wellbeing and reducing health inequalities. https://www.nice.org.uk/guidance/ng44/chapter/Committee-discussion#evidence.

[11] O’Mara-Eves A, Brunton G, McDaid D (2013). Community engagement to reduce inequalities in health: a systematic review, meta-analysis and economic analysis. Public Health Research.

[12] Stillwell D (2025). Comparing ethnicity data for different countries. https://dataingovernment.blog.gov.uk/2022/01/25/comparing-ethnicity-data-for-different-countries/.

[13] Office for National Statistics (2021). Ethnic group by religion: census. https://www.ons.gov.uk/datasets/RM031/editions/2021/versions/1.

[14] Bloom C (2023). Does Government ‘Do God?’: An Independent Review into How Government Engages with Faith.

[15] Zuriaga-Alvaro A, Kasstan-Dabush B, Johnson E (2025). Qualitative evaluation of two London Faith and Health Networks: lessons learnt from a model of an interface between health systems and minority communities. *BMJ Public Health*.

[16] Wehling H, Weston D, Hall C (2024). Use of UK faith Centre as a COVID-19 community vaccination clinic: exploring a potential model for community-based health care delivery. Postgrad Med J.

[17] All-Party Parliamentary Group on Faith and Society Keeping the faith: partnerships between faith groupsand local authorities during and beyond the pandemic. https://www.faithandsociety.org/keeping-the-faith/#:~:text=Keeping%20the%20Faith%20was%20published,during%20the%20COVID%2D19%20pandemic.

[18] Agboola J (2022). On Faith, Place and Health: Harnessing the power of faith groups to tackle London’s health inequalities.

[19] Hess S, Smith S, Umachandran S (2024). Faith as a complex system: engaging with the faith sector for strengthened health emergency preparedness and response. Lancet Glob Health.

[20] FaithAction (2014). The Impact of Faith-Based Organisations on Public Health and Social Capital.

[21] FaithAction, Local Government Association (2016). Working with Faith Groups to Promote Health and Wellbeing.

[22] Idler E, Jalloh MF, Cochrane J (2023). Religion as a social force in health: complexities and contradictions. BMJ.

[23] Ala A, Touray MML, Shafi S (2025). Harnessing faith-based organisations for global health equity. The Lancet.

[24] Ministry of Housing, Communities and Local Government COVID-19: guidance for the safe use of places of worship. https://www.gov.uk/government/publications/covid-19-guidance-for-the-safe-use-of-places-of-worship-during-the-pandemic-from-4-july/covid-19-guidance-for-the-safe-use-of-places-of-worship-from-2-december.

[25] Rozario M, Platt E (2025). Creating a Neighbourhood Health Service: The role of churches and faith groups in social prescribing.

[26] NHS England, Department of Health & Social Care (2022). Working in Partnership with People and Communities: Statutory Guidance.

[27] National Institute for Health and Care Research (2025). 23/149 faith-based groups and the impacts on health and health inequalities. https://www.nihr.ac.uk/documents/23149-faith-based-groups-and-the-impacts-on-health-and-health-inequalities/34654.

[28] Rai KK, Dogra SA, Barber S (2019). on behalf of the “Childhood Obesity Prevention in Islamic Religious Settings’ Programme Management Group”. A scoping review and systematic mapping of health promotion interventions associated with obesity in Islamic religious settings in the UK. Obes Rev.

[29] Bagnall A, South J, Trigwell J (2016). Community Engagement – Approaches to Improve Health: Map of the Literature on Current and Emerging Community Engagement Policy and Practice in the UK.

[30] Harden A, McKeown A, Dan-Ogosi I (2015). Evidence Review of Barriers to, and Facilitators of, Community Engagement Approaches and Practices in the UK.

[31] Munn Z, Peters MDJ, Stern C (2018). Systematic review or scoping review? Guidance for authors when choosing between a systematic or scoping review approach. BMC Med Res Methodol.

[32] Peters MDJ, Marnie C, Tricco AC (2020). Updated methodological guidance for the conduct of scoping reviews. *JBI Evid Synth*.

[33] Tricco AC, Lillie E, Zarin W (2018). PRISMA Extension for Scoping Reviews (PRISMA-ScR): Checklist and Explanation. Ann Intern Med.

[34] Waqar S, Yerrakalva D, Duffy TE (2024). Evaluation of a Faith-Placed Health Education Service on Bowel Cancer Screening in Mosques in East London. Health Expect.

[35] Kasstan B, Mounier-Jack S, Letley L (2022). Localising vaccination services: Qualitative insights on public health and minority group collaborations to co-deliver coronavirus vaccines. Vaccine (Auckl).

[36] Kasstan B, Mounier-Jack S, Zuriaga-Alvaro A (2023). “We’re potentially worsening health inequalities”: Evaluating how delivery of the 2022 London polio booster campaign was tailored to Orthodox Jewish families to reduce transmission vulnerability. *SSM Qual Res Health*.

[37] Codjoe L, N’Danga-Koroma J, Henderson C (2023). Pilot study of a manualised mental health awareness and stigma reduction intervention for Black faith communities in the UK: ON TRAC project. Soc Psychiatry Psychiatr Epidemiol.

[38] Christie-de Jong F, Kotzur M, Amiri R (2022). Qualitative evaluation of a codesigned faith-based intervention for Muslim women in Scotland to encourage uptake of breast, colorectal and cervical cancer screening. BMJ Open.

[39] Darko N, Dallosso H, Hadjiconstantinou M (2020). Qualitative evaluation of A Safer Ramadan, a structured education programme that addresses the safer observance of Ramadan for Muslims with Type 2 diabetes. Diabetes Res Clin Pract.

[40] King R, Warsi S, Amos A (2017). Involving mosques in health promotion programmes: a qualitative exploration of the MCLASS intervention on smoking in the home. Health Educ Res.

[41] Maynard M, Baker G, Harding S (2017). Exploring childhood obesity prevention among diverse ethnic groups in schools and places of worship: Recruitment, acceptability and feasibility of data collection and intervention components. Prev Med Rep.

[42] Shah S, Ainsworth H, Fairhurst C (2015). Muslim communities learning about second-hand smoke: a pilot cluster randomised controlled trial and cost-effectiveness analysis. NPJ Prim Care Respir Med.

[43] Mantovani N, Pizzolati M, Gillard S (2017). Engaging communities to improve mental health in African and African Caribbean groups: a qualitative study evaluating the role of community well-being champions. *Health Soc Care Community*.

[44] Hall J, Rashid R, Rafiq A (2024). Reflections on co-producing an obesity-prevention toolkit for Islamic Religious Settings: a qualitative process evaluation. Int J Behav Nutr Phys Act.

[45] Ali OME, Gkekas E, Ali AMS (2023). Informing the UK Muslim Community on Organ Donation: Evaluating the Effect of a National Public Health Programme by Health Professionals and Faith Leaders. J Relig Health.

[46] Perry A, Gardener C, Dove J (2018). Improving mental health knowledge of the Charedi Orthodox Jewish Community in North London: A partnership project. Int J Soc Psychiatry.

[47] Kotzur M, Amiri R, Gatting L (2023). Adapting Participatory Workshops to a Virtual Setting: Co-Design With Muslim Women of a Faith-Based Intervention to Encourage Cancer Screening Uptake. Int J Qual Methods.

[48] Hankir A, Khalil S, Wadood Q (2017). The Federation of Student Islamic Societies programme to challenge mental health Stigma in Muslim communities in England: The FOSIS Birmingham study. Psychiatr Danub.

[49] All-Party Parliamentary Group on Faith and Society Keeping the faith 2.0: embedding a new normal for partnership working in post-pandemic britain. https://research.gold.ac.uk/id/eprint/32173/.

[50] Tomalin E, Sadgrove J, Summers R (2019). Health, faith and therapeutic landscapes: Places of worship as Black, Asian and Minority Ethnic (BAME) public health settings in the United Kingdom. *Social Science & Medicine*.

