# Supplementary material for: Engaging with faith communities to tackle ethnic health inequalities in the UK: a scoping review
Source: BMJ Public Health. 2026 Jan 27;4(1):e003816. doi: 10.1136/bmjph-2025-003816 (PMC12853493; doi:10.1136/bmjph-2025-003816)
Supplement: online supplemental appendix 1 [file bmjph-4-1-s001.docx]

**Appendix 1: Inclusion and exclusion criteria used in the scoping review**

| Category | Inclusion Criteria | Exclusion Criteria |
| --- | --- | --- |
| *Population* | - Studies of minoritised ethnic groups in the UK, defined as all ethnic groups excluding the White British ^1^. According to the 2021 England and Wales census ^2^, this includes Asian, Asian British or Asian Welsh; Black, Black British, Black Welsh, Caribbean or African; Mixed or Multiple ethnic groups; White Irish; White Gypsy, Irish Travellers, Roma or Other White; and Other ethnic group). - We also include studies of Jewish and Sikh groups as they are legally recognised as ethno-religious groups under the UK Equality Act 2010 ^3^. | - Studies without a focus on ethnic minorities, or where ethnicity, religion, or language is not specified or relevant. |
| *Concept* | - Studies of public health interventions involving faith CE in their design or delivery, with health outcomes related to EHIs or outcomes based on social and structural determinants of a named EHI (e.g. social support or racism). - Studies exploring experiences, including barriers and facilitators, of engaging faith communities in designing or delivering public health interventions. | - Studies with no meaningful involvement of faith communities, such as studies where faith settings have been used without any faith community involvement - Studies without identifiable outcomes or experiences linked to identifiable faith CE. |
| *Context* | - Any geographical setting in the UK. Including England/Wales/Scotland/Northern Ireland; including devolved administrations and local authority programs | - Studies outside of the UK or where UK-specific data cannot be extracted. |
| *Study Design* | Empirical studies only. They could be evaluations or process studies, including   - Quantitative: Any studies assessing outcomes using quantitative study designs including randomised controlled studies, quasi-experimental, before-and-after, cohort, cross-sectional, case-control studies. - Qualitative: Any studies using qualitative methods including interviews, focus groups, ethnography, participatory approaches and surveys. - Mixed methods: Included if either qualitative or quantitative component meets inclusion criteria. - Systematic reviews: Included only to identify eligible primary studies.   Student theses are included if they meet the above criteria | - Non-empirical studies (e.g., opinion pieces or commentaries). - Abstracts with no full text. Purely descriptive reports (e.g., reporting attendance or number of tests only without analysis of outcome or experience of faith CE. |
| *Timeframe* | - Studies published between June 2014 – August 2024 inclusive**.** | - Published before June 2014. |
| *Language* | English-language publications only. | Non-English publications. |

References

(1) Race Disparity Unit. *Writing about ethnicity.* <https://www.ethnicity-facts-figures.service.gov.uk/style-guide/writing-about-ethnicity> [Accessed July 2025].

(2) Office for National Statistics. *Ethnic group by religion: Census 2021.* <https://www.ons.gov.uk/datasets/RM031/editions/2021/versions/1> [Accessed July 2025].

(3) *Equality Act 2010, c.15.* [Internet]. London: The Stationery Office; 2010. Available from: <https://www.legislation.gov.uk/ukpga/2010/15/contents>. [Accessed July 2025].
