# Supplementary material for: Engaging with faith communities to tackle ethnic health inequalities in the UK: a scoping review
Source: BMJ Public Health. 2026 Jan 27;4(1):e003816. doi: 10.1136/bmjph-2025-003816 (PMC12853493; doi:10.1136/bmjph-2025-003816)
Supplement: online supplemental appendix 2 [file bmjph-4-1-s002.docx]

**Ovid MEDLINE(R) ALL <1946 to August 26, 2024>**

1 *health education/ or exp health fairs/ or exp health promotion/ or exp patient education as topic/ 206055

2 (Intiative* or scheme* or project* or interven* or campaign* or course* or program* or outreach* or "health adj5 improv*" or "health* adj4 fair*" or champion* or "health adj4 promot*" or "health adj5 educat*" or preven* or wellness or well?being or "health* adj3 check*" or "behavio?r" or "health* adj4 aware*" or uptake or "public adj3 health" or collab* or co?deliver* or screen* or deliver* or "risk adj3 assess*" or pilot* or trial or co?design* or engag*).ti,ab. 8424233

3 1 or 2 8514038

4 exp Religion/ 68108

5 exp Faith-Based Organizations/ 1812

6 (Relig* or Spiritual* or Church* or Mosque* or Faith* or Christian* or Islam* or Muslim* or temple* or hindu* or judaism* or jew* or Sikh* or gurdwara* or synagogue* or masjid* or mandir* or jinja* or "worship" or cathedral* or parish* or pastor* or imam* or priest* or clergy* or chaplain*).ti,ab. 147220

7 4 or 5 or 6 181802

8 exp United Kingdom/ 397850

9 ("united kingdom" or uk or England or "northern ireland" or scotland or Wales or Britain or NHS).mp. 565298

10 8 or 9 581269

11 exp "Ethnic and Racial Minorities"/ 1084

12 exp Minority Groups/ 18898

13 exp Minority Health/ 908

14 "Health Disparate Minority and Vulnerable Populations"/ 41

15 (African* or Caribbean* or mixed or Black* or Afr* Caribbean* or Minorit* or Ethnic* or Race* or raci* or colo?r or BAME or BME or Asia* or Bangladeshi* or Indian* or Pakistani* or Arab* or Roma* or Irish* or gyps* or Chinese*).mp. 2462052

16 11 or 12 or 13 or 14 or 15 2462052

17 3 and 7 and 10 and 16 770

18 limit 17 to yr="2014 - 2024" 417

**Embase Classic+Embase <1947 to 2024 August 26>**

1 exp health program/ or exp health promotion/ or exp health promotion model/ or exp health risk assessment/ 272473

2 (Intiative* or scheme* or project* or interven* or campaign* or course* or program* or outreach* or "health adj5 improv*" or "health* adj4 fair*" or champion* or "health adj4 promot*" or "health adj5 educat*" or preven* or wellness or well?being or "health* adj3 check*" or "behavio?r" or "health* adj4 aware*" or uptake or "public adj3 health" or collab* or co?deliver* or screen* or deliver* or "risk adj3 assess*" or pilot* or trial or co?design* or engag*).ti,ab. 11415007

3 1 or 2 11500168

4 exp religion/ or exp religiosity/ 87993

5 exp faith-based organization/ 337

6 (Relig* or Spiritual* or Church* or Mosque* or Faith* or Christian* or Islam* or Muslim* or temple* or hindu* or judaism* or jew* or Sikh* or gurdwara* or synagogue* or masjid* or mandir* or jinja* or "worship" or cathedral* or parish* or pastor* or imam* or priest* or clergy* or chaplain*).ti,ab. 188224

7 4 or 5 or 6 223973

8 exp United Kingdom/ 513444

9 ("united kingdom" or uk or England or "northern ireland" or scotland or Wales or Britain or NHS).mp. 970904

10 8 or 9 970904

11 exp ethnic difference/ or exp ethnic background/ or exp ethnic identity/ or exp ethnic group/ 243624

12 exp minority group/ 66595

13 exp racial disparity/ or exp racial segregation/ or exp racial background/ or exp racial identity/ or exp racial diversity/ 7984

14 exp race/ or exp race relation/ 113118

15 exp vulnerable population/ 31214

16 (African* or Caribbean* or mixed or Black* or Afr* Caribbean* or Minorit* or Ethnic* or Race* or raci* or colo?r or BAME or BME or Asia* or Bangladeshi* or Indian* or Pakistani* or Arab* or Roma* or Irish* or gyps* or Chinese*).mp. 3363466

17 11 or 12 or 13 or 14 or 15 or 16 3491542

18 3 and 7 and 10 and 17 1255

19 limit 18 to yr="2014 - 2024" 726

**APA PsycInfo <1806 to August 2024 Week 4>**

1 exp health promotion/ or exp health risk behavior/ or exp health screening/ 54511

2 (Intiative* or scheme* or project* or interven* or campaign* or course* or program* or outreach* or "health adj5 improv*" or "health* adj4 fair*" or champion* or "health adj4 promot*" or "health adj5 educat*" or preven* or wellness or well?being or "health* adj3 check*" or "behavio?r" or "health* adj4 aware*" or uptake or "public adj3 health" or collab* or co?deliver* or screen* or deliver* or "risk adj3 assess*" or pilot* or trial or co?design* or engag*).ti,ab. 2270595

3 1 or 2 2281451

4 exp religion/ or exp "religion and spirituality measures"/ or exp religiosity/ or exp religious affiliation/ or exp religious beliefs/ or exp religious buildings/ 98100

5 exp faith/ or exp faith based organizations/ 4264

6 (Relig* or Spiritual* or Church* or Mosque* or Faith* or Christian* or Islam* or Muslim* or temple* or hindu* or judaism* or jew* or Sikh* or gurdwara* or synagogue* or masjid* or mandir* or jinja* or "worship" or cathedral* or parish* or pastor* or imam* or priest* or clergy* or chaplain*).ti,ab. 161816

7 4 or 5 or 6 182455

8 ("united kingdom" or uk or England or "northern ireland" or scotland or Wales or Britain or NHS).mp. 120069

9 exp ethnic diversity/ or exp ethnic identity/ or exp ethnic values/ 23024

10 exp "race and ethnic discrimination"/ or exp "race (anthropological)"/ or exp "racial and ethnic attitudes"/ 34071

11 (African* or Caribbean* or mixed or Black* or Afr* Caribbean* or Minorit* or Ethnic* or Race* or raci* or colo?r or BAME or BME or Asia* or Bangladeshi* or Indian* or Pakistani* or Arab* or Roma* or Irish* or gyps* or Chinese*).mp. 679720

12 9 or 10 or 11 680758

13 3 and 7 and 8 and 12 690

14 limit 13 to yr="2014 - 2024" 353

**SCOPUS**

( TITLE-ABS-KEY ( intiative* OR scheme* OR project* OR interven* OR campaign* OR course* OR program* OR outreach* OR "health adj5 improv*" OR "health* adj4 fair*" OR champion* OR "health adj4 promot*" OR "health adj5 educat*" OR preven* OR wellness OR well?being OR "health* adj3 check*" OR "behavio?r" OR "health* adj4 aware*" OR uptake OR "public adj3 health" OR collab* OR co?deliver* OR screen* OR deliver* OR "risk adj3 assess*" OR pilot* OR trial OR co?design* OR engag* ) AND TITLE-ABS-KEY ( "united kingdom" OR uk OR england OR "northern ireland" OR scotland OR wales OR britain OR nhs ) AND TITLE-ABS-KEY ( relig* OR spiritual* OR church* OR mosque* OR faith* OR christian* OR islam* OR muslim* OR temple* OR hindu* OR judaism* OR jew* OR sikh* OR gurdwara* OR synagogue* OR masjid* OR mandir* OR jinja* OR "worship" OR cathedral* OR parish* OR pastor* OR imam* OR priest* OR clergy* OR chaplain* ) AND TITLE-ABS-KEY ( african* OR caribbean* OR mixed OR black* OR afr* AND caribbean* OR minorit* OR ethnic* OR race* OR raci* OR colo?r OR bame OR bme OR asia* OR bangladeshi* OR indian* OR pakistani* OR arab* OR roma* OR irish* OR gyps* OR chinese* ) ) AND PUBYEAR > 2013 AND PUBYEAR < 2025 AND ( LIMIT-TO ( AFFILCOUNTRY , "United Kingdom" ) )

**Web of Science Search Strategy**

1: TS=(Intiative* OR scheme* OR project* OR interven* OR campaign* OR course* OR program* OR outreach* OR "health NEAR5 improv*" OR "health* NEAR4 fair*" OR champion* OR "health NEAR4 promot*" OR "health NEAR5 educat*" OR preven* OR wellness OR well?being OR "health* NEAR3 check*" OR "behavio?r" OR "health* NEAR4 aware*" OR uptake OR "public NEAR3 health" OR collab* OR co?deliver* OR screen* OR deliver* OR "risk NEAR3 assess*" OR pilot* OR trial OR co?design* OR engag*) Date Run: Wed Aug 28 2024 22:01:42 GMT+0100 (British Summer Time) Results: 15068122

2: TS=(Relig* or Spiritual* or Church* or Mosque* or Faith* or Christian* or Islam* or Muslim* or temple* or hindu* or judaism* or jew* or Sikh* or gurdwara* or synagogue* or masjid* or mandir* or jinja* or "worship" or cathedral* or parish* or pastor* or imam* or priest* or clergy* or chaplain*) and Religion (Should – Search within topic) and Spirituality (Should – Search within topic) and Islam (Should – Search within topic) and Religiosity (Should – Search within topic) and Christianity (Should – Search within topic) and Church (Should – Search within topic) and Faith (Should – Search within topic) and Muslims (Should – Search within topic) and Jews (Should – Search within topic) Date Run: Wed Aug 28 2024 22:09:39 GMT+0100 (British Summer Time) Results: 778232

3: TS=("united kingdom" or uk or England or "northern ireland" or scotland or Wales or Britain or NHS) and United Kingdom (Should – Search within topic) and Uk (Should – Search within topic) and Scotland (Should – Search within topic) and England (Should – Search within topic) and Britain (Should – Search within topic) and Northern Ireland (Should – Search within topic) and Great Britain (Should – Search within topic) and Wales (Should – Search within topic) Date Run: Wed Aug 28 2024 22:10:15 GMT+0100 (British Summer Time) Results: 726470

4: TS=(African* or Caribbean* or mixed or Black* or Afr* Caribbean* or Minorit* or Ethnic* or Race* or raci* or colo?r or BAME or BME or Asia* or Bangladeshi* or Indian* or Pakistani* or Arab* or Roma* or Irish* or gyps* or Chinese*) and Ethnicity (Should – Search within topic) and Race (Should – Search within topic) and Racism (Should – Search within topic) Date Run: Wed Aug 28 2024 22:10:51 GMT+0100 (British Summer Time) Results: 5431117

5: #4 AND #3 AND #2 AND #1 Date Run: Wed Aug 28 2024 22:11:29 GMT+0100 (British Summer Time) Results: 1820

6: #4 AND #3 AND #2 AND #1 and 2024 or 2023 or 2022 or 2021 or 2020 or 2019 or 2018 or 2017 or 2016 or 2015 or 2014 (Publication Years) Date Run: Wed Aug 28 2024 22:11:55 GMT+0100 (British Summer Time) Results: 1235

7: #4 AND #3 AND #2 AND #1 and 2024 or 2023 or 2022 or 2021 or 2020 or 2019 or 2018 or 2017 or 2016 or 2015 or 2014 (Publication Years) and ENGLAND or SCOTLAND or NORTH IRELAND or WALES (Countries/Regions) Date Run: Wed Aug 28 2024 22:12:19 GMT+0100 (British Summer Time) Results: 753

**CINAHL through Ebsco Search Strategy**

Select / deselect all

| [**Search ID#**](javascript:__doPostBack('ctl00$ctl00$FindField$FindField$historyControl$ReorderHistoryLink','')) | | **Search Terms** |
| --- | --- | --- |
|  | S16 | S3 AND S6 AND S9 AND S13 |
|  | S15 | S3 AND S6 AND S9 AND S13 |
|  | S14 | S3 AND S6 AND S9 AND S13 |
|  | S13 | S10 OR S11 OR S12 |
|  | S12 | African* or Caribbean* or mixed or Black* or Afr* Caribbean* or Minorit* or Ethnic* or Race* or raci* or colo?r or BAME or BME or Asia* or Bangladeshi* or Indian* or Pakistani* or Arab* or Roma* or Irish* or gyps* or Chinese* |
|  | S11 | (MH "Racism+") OR (MM "Racialization") OR (MH "Discrimination+") |
|  | S10 | (MH "Minority Groups+") OR (MH "Ethnic Groups+") OR (MH "Health Personnel, Minority+") OR (MM "Racialization") OR (MM "Racial Equality") |
|  | S9 | S7 OR S8 |
|  | S8 | united kingdom or uk or britain or scotland or england or wales or northern ireland |
|  | S7 | (MH "Great Britain+") OR (MH "United Kingdom+") OR (MM "Ireland") OR (MM "Scotland") OR (MM "England") OR (MM "Wales") OR (MM "Northern Ireland") |
|  | S6 | S4 OR S5 |
|  | S5 | Relig* or Spiritual* or Church* or Mosque* or Faith* or Christian* or Islam* or Muslim* or temple* or hindu* or judaism* or jew* or Sikh* or gurdwara* or synagogue* or masjid* or mandir* or jinja* or "worship" or cathedral* or parish* or pastor* or imam* or priest* or clergy* or chaplain*). |
|  | S4 | (MH "Religion and Religions+") OR (MM "Religion and Medicine") OR (MH "Named Groups by Religion+") OR (MM "Faith-Based Organizations") OR (MH "Faith Community Nurses+") OR (MH "Faith Community Nursing+") OR (MM "Muslims") OR (MM "Psychological Well-Being") OR (MH "Orthodox Jews+") OR (MM "Sikhs") OR (MM "Monks") OR (MM "Nuns") OR (MH "Jews+") OR (MM "Hinduism") OR (MH "Christianity+") OR (MM "Clergy") OR (MM "Chaplains") OR (MM "Buddhism") OR (MM "Catholicism") OR (MM "Chaplaincy Service, Hospit [...](javascript:showHistoryTerm('ctl00_ctl00_FindField_FindField_historyControl_HistoryRepeater_ctl12_ellipsis',true)) |
|  | S3 | S1 OR S2 |
|  | S2 | Intiative* or scheme* or project* or interven* or campaign* or course* or program* or outreach* or "health adj5 improv*" or "health* adj4 fair*" or champion* or "health adj4 promot*" or "health adj5 educat*" or preven* or wellness or well?being or "health* adj3 check*" or "behavio?r" or "health* adj4 aware*" or uptake or "public adj3 health" or collab* or co?deliver* or screen* or deliver* or "risk adj3 assess*" or pilot* or trial or co?design* or engag* |
|  | S1 | (MH "Health Promotion+") OR (MH "Health Behavior+") OR (MH "Health Promoting Behavior (Iowa NOC)") OR (MH "Education, Health Information Management") OR (MH "Mental Health Promotion (Saba CCC)") OR (MH "Health Seeking Behavior Alteration (Saba CCC)") OR (MH "Oral Health Promotion (Iowa NIC)") OR (MM "Life Style Changes") OR (MH "Health Promotion (Saba CCC)") OR (MH "Health Behavior (Iowa NOC)") OR (MH "Health Seeking Behaviors (NANDA)") OR (MH "Royal Society for the Promotion of Health") OR (MH  [...](javascript:showHistoryTerm('ctl00_ctl00_FindField_FindField_historyControl_HistoryRepeater_ctl15_ellipsis',true)) |

**Cochrane Library Search Strategy**

ID Search

#1 MeSH descriptor: [Health Promotion] explode all trees

#2 (Intiative* or scheme* or project* or interven* or campaign* or course* or program* or outreach* or "health adj5 improv*" or "health* adj4 fair*" or champion* or "health adj4 promot*" or "health adj5 educat*" or preven* or wellness or well?being or "health* adj3 check*" or "behavio?r" or "health* adj4 aware*" or uptake or "public adj3 health" or collab* or co?deliver* or screen* or deliver* or "risk adj3 assess*" or pilot* or trial or co?design* or engag*):ti,ab,kw (Word variations have been searched)

#3 #1 or #2

#4 MeSH descriptor: [Faith-Based Organizations] explode all trees

#5 MeSH descriptor: [Religion] this term only

#6 (Relig* or Spiritual* or Church* or Mosque* or Faith* or Christian* or Islam* or Muslim* or temple* or hindu* or judaism* or jew* or Sikh* or gurdwara* or synagogue* or masjid* or mandir* or jinja* or "worship" or cathedral* or parish* or pastor* or imam* or priest* or clergy* or chaplain*):ti,ab,kw

#7 MeSH descriptor: [United Kingdom] explode all trees

#8 ("united kingdom" or uk or England or "northern ireland" or scotland or Wales or Britain or NHS):ti,ab,kw

#9 #4 or #5 or #6

#10 #7 or #8

#11 MeSH descriptor: [Health Inequities] explode all trees

#12 MeSH descriptor: [Racism] explode all trees

#13 MeSH descriptor: [Ethnic and Racial Minorities] explode all trees

#14 (African* or Caribbean* or mixed or Black* or Afr* Caribbean* or Minorit* or Ethnic* or Race* or raci* or colo?r or BAME or BME or Asia* or Bangladeshi* or Indian* or Pakistani* or Arab* or Roma* or Irish* or gyps* or Chinese*):ti,ab,kw

#15 #11 or #12 or #13 or #14

#16 #3 AND #9 AND #10 AND #15 with Cochrane Library publication date Between Jun 2014 and Aug 2024

**HMIC Health Management Information Consortium <1979 to July 2024>**

1 exp health programmes/ or exp health promotion/ or exp health promotion officers/ 12436

2 (Intiative* or scheme* or project* or interven* or campaign* or course* or program* or outreach* or "health adj5 improv*" or "health* adj4 fair*" or champion* or "health adj4 promot*" or "health adj5 educat*" or preven* or wellness or well?being or "health* adj3 check*" or "behavio?r" or "health* adj4 aware*" or uptake or "public adj3 health" or collab* or co?deliver* or screen* or deliver* or "risk adj3 assess*" or pilot* or trial or co?design* or engag*).ti,ab. 124781

3 1 or 2 130190

4 exp religion/ or exp religion & belief equality/ or exp religion & society/ or exp religions/ or exp religious activities/ 526

5 (Relig* or Spiritual* or Church* or Mosque* or Faith* or Christian* or Islam* or Muslim* or temple* or hindu* or judaism* or jew* or Sikh* or gurdwara* or synagogue* or masjid* or mandir* or jinja* or "worship" or cathedral* or parish* or pastor* or imam* or priest* or clergy* or chaplain*).ti,ab. 2712

6 4 or 5 2849

7 exp United Kingdom/ 47496

8 ("united kingdom" or uk or England or "northern ireland" or scotland or Wales or Britain or NHS).mp. 131661

9 7 or 8 146350

10 exp ethnic differences/ or exp ethnic diversity/ or exp ethnic groups/ or exp ethnic minorities/ or exp ethnic monitoring/ or exp ethnicity/ 6250

11 exp race relations/ 739

12 exp racial discrimination/ or exp racial equality/ or exp racial inequality/ 988

13 (African* or Caribbean* or mixed or Black* or Afr* Caribbean* or Minorit* or Ethnic* or Race* or raci* or colo?r or BAME or BME or Asia* or Bangladeshi* or Indian* or Pakistani* or Arab* or Roma* or Irish* or gyps* or Chinese*).mp. 21458

14 10 or 11 or 12 or 13 21903

15 3 and 6 and 9 and 14 203

16 limit 15 to yr="2014 - 2024" 33
