# Supplementary material for: Engaging with faith communities to tackle ethnic health inequalities in the UK: a scoping review
Source: BMJ Public Health. 2026 Jan 27;4(1):e003816. doi: 10.1136/bmjph-2025-003816 (PMC12853493; doi:10.1136/bmjph-2025-003816)
Supplement: online supplemental appendix 3 [file bmjph-4-1-s003.docx]

**DATA EXTRACTION TEMPLATE**

**Lead author, Year**

**Title**

**Aim**

**Publication Year**

**Country/city**

**Study design**

1. Randomised controlled trial
2. Non-randomised experimental study
3. Cohort study
4. case-control study
5. Systematic review
6. Qualitative research
7. Mixed methods study
8. Other

**Sample size/age/sex**

**Ethnic minority group targeted**

1. Asian, Asian British or Asian Welsh
2. Black, Black British, Black Welsh, Caribbean or African
3. Mixed or Multiple ethnic groups
4. White: Irish
5. White: Gypsy or Irish Traveller, Roma or Other White
6. Other ethnic group

**What is the primary health inequality being targeted?**

1. Infectious disease
2. Cardiovascular disease
3. Diabetes
4. Cancer
5. Respiratory disease
6. Depression or Anxiety
7. Psychosis
8. Maternal health
9. Dementia and Alzheimer's
10. Other mental health
11. Renal disease
12. Haematological disease
13. Neurological disease
14. Obesity
15. Diet
16. Physical activity
17. Smoking
18. Alcohol
19. Screening
20. Other

**Faith community engaged**

1. Christian
2. Muslim
3. Buddhist
4. Hindu
5. Jewish
6. Sikh
7. None
8. Unknown
9. Other

**What strategy is used by the public health intervention?**

1. Create supportive environments (apart from place of worship)
2. Health education e.g through a campaign
3. Building healthy public policy
4. Prevention: Routine national screening
5. Prevention: Other early detection
6. Counselling/behaviour change advice
7. Immunisation
8. Other disease prevention
9. None

**Was the intervention designed or delivered locally, regionally or nationally?**

**State briefly what the public health intervention was.**

**Which faith community engagement approach(es) is/are used?**

1. Strengthening communities
2. Volunteer and Peer roles
3. Collaborations and Partnerships
4. Access to community resources

**Give a description of the faith community engagement approach(es).**

**Which health system actors were involved?**

**What were the roles of the health system actors involved?**

**What are the barriers to faith community engagement?**

**What are the facilitators of faith community engagement?**

**What type of health outcome are reported?**

***Health behaviour***

1. Smoking
2. Alcohol
3. Physical activity
4. Healthy eating.
5. Cancer screening
6. Immunisation uptake
7. Other screening uptake
8. Precursor eg knowledge, self-efficacy
9. Other Health behaviour

***Physical health***

1. Obesity/Weight
2. Diabetes
3. Stroke
4. Cardiovascular disease
5. Hypertension
6. Unintended consequences
7. Infectious disease
8. None
9. Unknown

***Mental Health***

***social outcomes***

1. Capacity building
2. Social capital
3. Increased community resources
4. Changes to physical, social or economic environment
5. None
6. Unknown
7. Other

***Other wider determinants***

***-***Racism, housing etc

**Describe the outcome**

**What are other important findings?**
